# Staphylococcus aureus (S. aureus) Lobar Pneumonia Following Bronchoscopic Lung-Volume Reduction (BLVR): A Case Report and Review of Literature

**DOI:** 10.7759/cureus.25579

**Published:** 2022-06-01

**Authors:** Mansur Assaad, Neha Siddiqui, Faraz Siddiqui, Boyd Hehn

**Affiliations:** 1 Pulmonary and Critical Care Medicine, Guthrie Robert Packer Hospital, Sayre, USA; 2 Biomedical Sciences, University College Dublin School of Medicine, Dublin, IRL

**Keywords:** copd, post-procedure complication, bacterial pneumonia, endobronchial valve, bronchoscopic lung volume reduction

## Abstract

With the advent of bronchoscopic lung-volume reduction (BLVR), this minimally invasive technique represents a new and effective way of managing the debilitating symptoms associated with severe centrilobular emphysema. Despite its vast potential in the management of this disease, there are still several potential risk factors associated with the procedure that may predispose the patient to increased morbidity. Our patient received four endobronchial valves in the right-upper lobe (RUL) and right-middle lobe (RML). Although her immediate post-procedure course was uncomplicated, she returned shortly after discharge with a right-sided pneumothorax and right-lower lobar pneumonia with sputum culture growing methicillin-sensitive *Staphylococcus aureus* (*S. aureus*). She was managed with tube thoracostomy and two weeks of cefazolin with clinical improvement. Despite the abundance of literature detailing the risk of pneumonia following BLVR, very little data exists discussing common causative organisms, choice of treatment, duration of treatment, and potential risk factors that may predispose these patients to infection.

## Introduction

Bronchoscopic lung-volume reduction (BLVR) has been developed for the management of patients with severe, heterogenous emphysema with persistent symptoms despite standard medical therapy [1]. The placement of a one-way endobronchial valves in the diseased, collateral ventilation-negative lobe allows air to empty and prevents air re-entry, thus reducing hyperinflation and improving the work of breathing [1]. Several randomized control trials have proven the efficacy of BLVR in improving symptom-related quality of life, exercise capacity, and lung function [1,2].

Despite the significant benefit provided by BLVR, there are several potential complications that patients can suffer following valve implantation. Such potential complications include pneumothorax, acute exacerbation of chronic obstructive pulmonary disease (COPD), valve migration, bronchial torsion, hemoptysis, pneumonia, and even death [1,2]. A systematic review by Gülşen in 2018 reported the incidence of pneumonia following BLVR to be between 3% and 11.7% [1]. However, little data exists overall regarding pneumonia following BLVR, specifically factors such as causative organism, choice of antibiotics, duration of antibiotics, and predisposing risk factors such as aspiration or concomitant use of inhaled corticosteroids (ICS). Herein, we present a case of necrotizing pneumonia from *Staphylococcus aureus* (*S. aureus*) following BLVR that was successfully treated with two weeks of antibiotics.

## Case presentation

**Figure 1:**
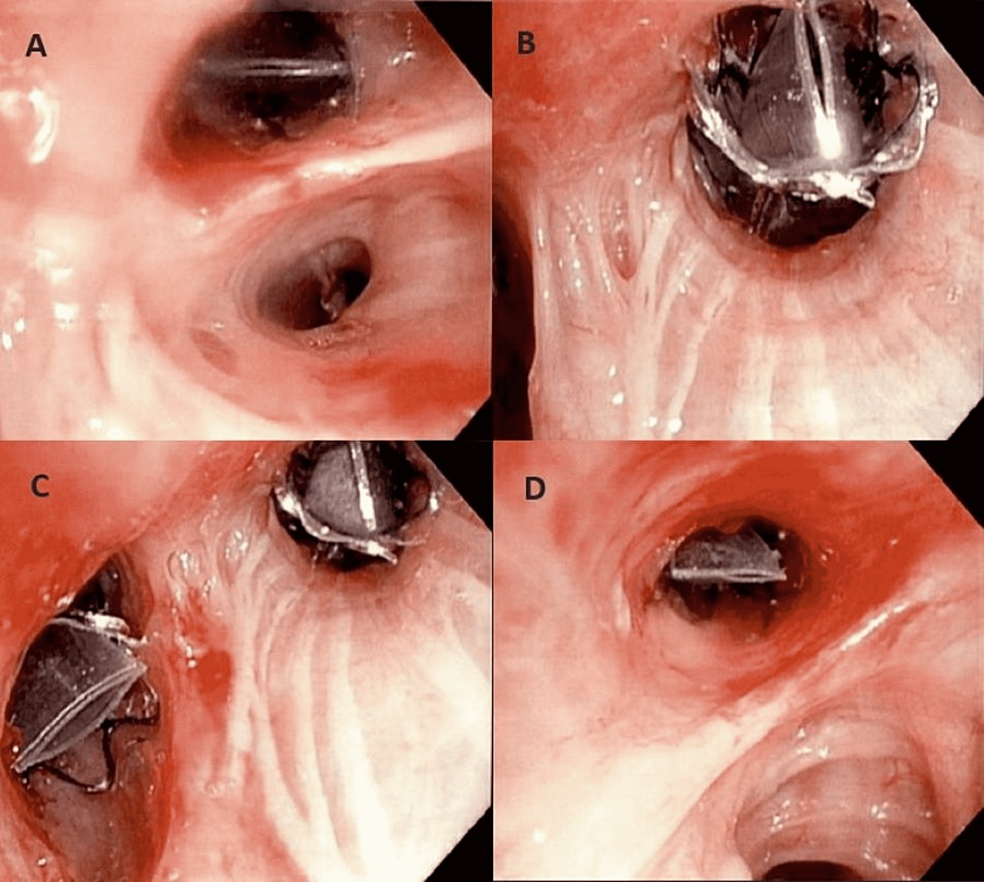
Post BLVR showing size 4-LP in RB 1 (A); size 4-LP in RB2 (B); size 5.5 in RB3 (C); and size 5.5 in RB4/5 (D) BLVR: bronchoscopic lung-volume reduction; LP: low profile; RB: right bronchus

A 65-year-old woman with severe emphysema (forced expiratory volume at 1 second (FEV1) 28% of predicted) on maximum inhalation therapy with combination fluticasone/umeclidinium/vilanterol 100/62.5/25 mcg daily was admitted following routine BLVR of the right-upper lobe (RUL) and right-middle lobe (RML). A total of four Zephyr® endobronchial valves (Pulmonx, 2021) were placed: size 4-low profile (LP) in right bronchus 1 (RB1), size 4-LP in RB2, size 5.5 in RB3, and size 5.5 in RB4/5 (Figure 1).

**Figure 2:**
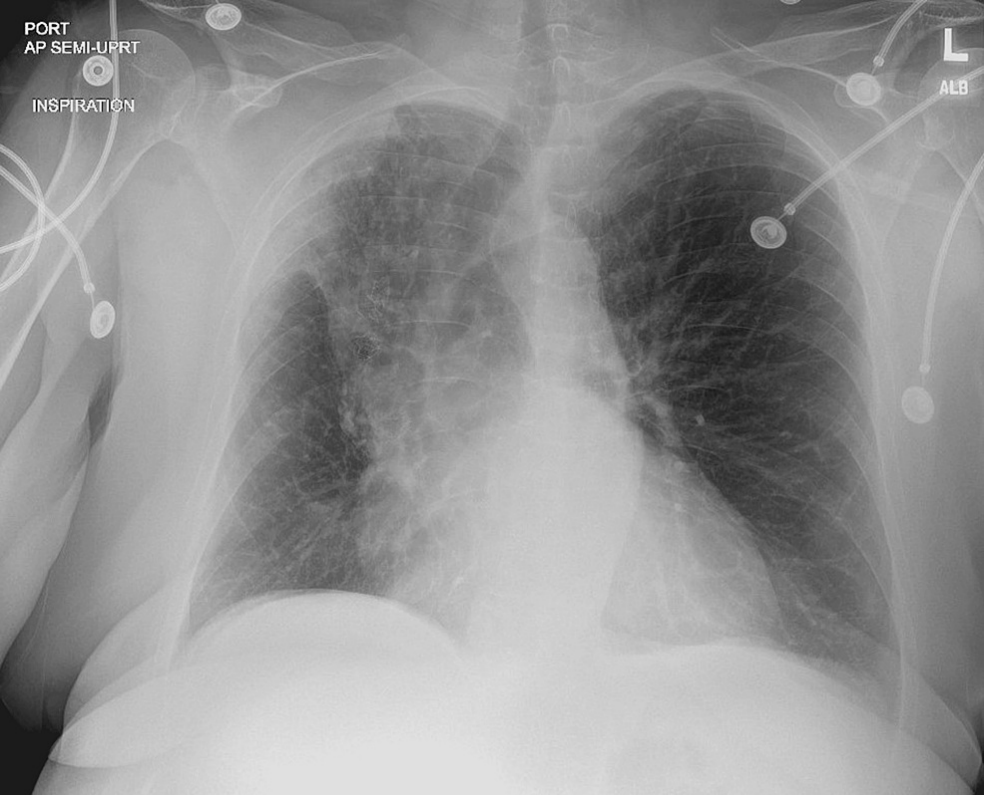
Post-procedure chest radiograph showing rapid atelectasis of the RUL and tracheal deviation to the right RUL: right-upper lobe

A chest radiograph following the procedure showed early atelectatic changes and no pneumothorax (Figure 2).

**Figure 3:**
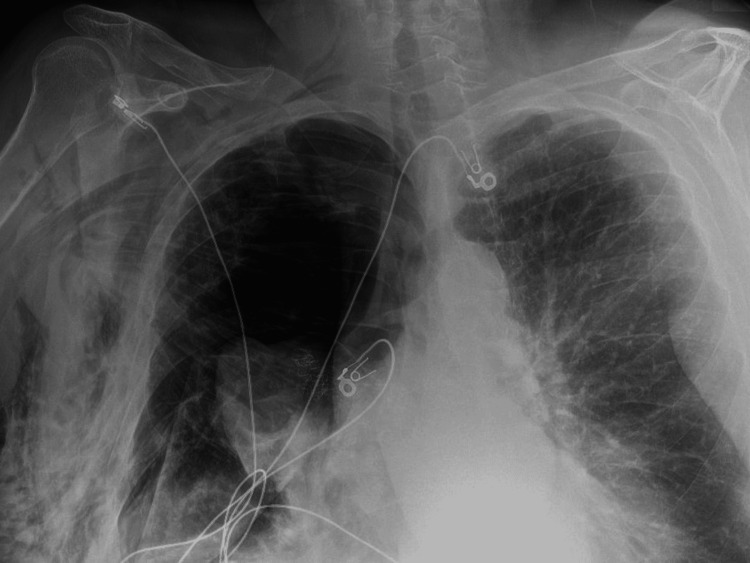
Chest radiograph showing right-sided pneumothorax following removal of malpositioned chest tube

The patient suffered no immediate complications and was discharged home in stable condition four days after the procedure. She returned to the emergency department six days later in severe respiratory distress. She underwent needle thoracostomy for empiric treatment of suspected tension pneumothorax followed by insertion of a 20-French chest tube on the right. Computed tomography (CT) showed the tube to be sub-pleural, so it was removed due to malposition. Follow-up radiograph showed clear evidence of pneumothorax (Figure 3).

**Figure 4:**
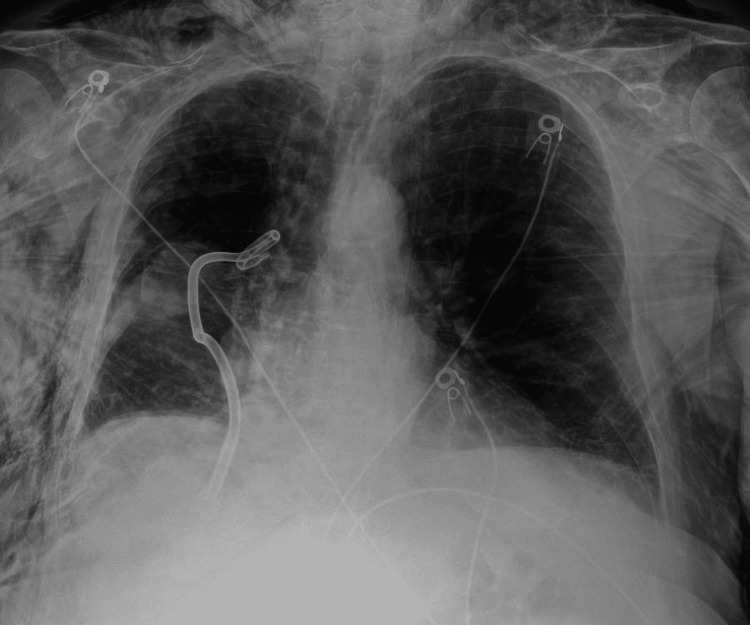
Chest radiograph showing persistent right pneumothorax despite placement of a 14-French pigtail catheter.

A 14-French pigtail catheter was placed by interventional radiology, with no significant improvement in the pneumothorax (Figure 4).

**Figure 5:**
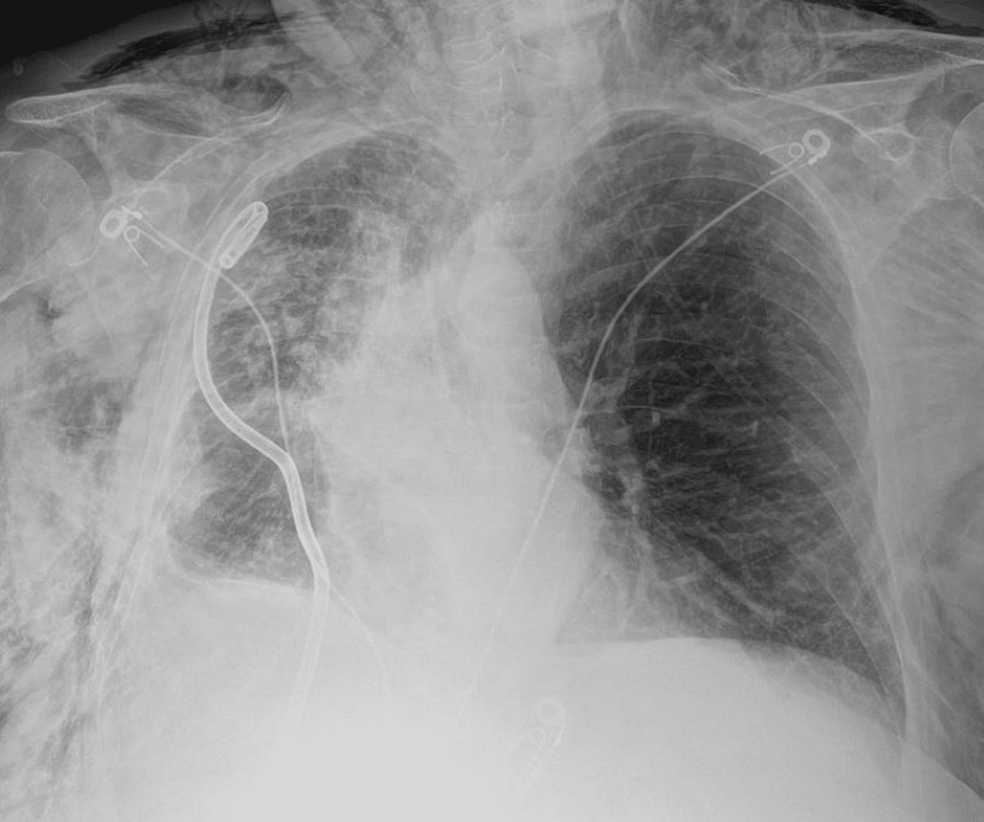
Chest radiograph following explantation of the RML valve resulting in significant lung re-expansion and resolution of continuous air leak RML:

There was a continuous air leak from the chest tube, so the patient underwent urgent repeat bronchoscopy with explantation of the RML valve for suspected bronchopleural fistula. Following the procedure, the air leak remained 1+ and intermittent, and repeat radiograph showed adequate lung re-expansion (Figure 5).

**Figure 6:**
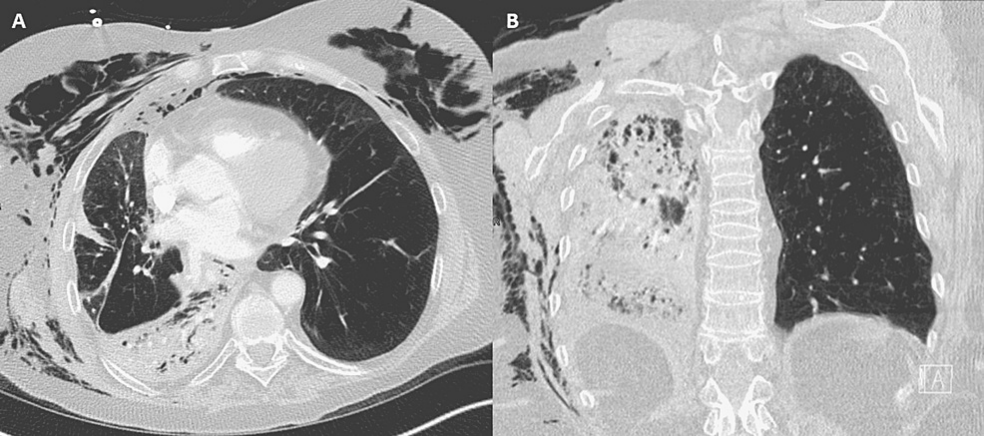
Axial (A) and coronal (B) chest CT images showing a dense right-lower lobar pneumonia. Extensive subcutaneous emphysema is also present. CT: computed tomography

Despite this intervention, the patient’s hypoxia worsened to the point of requiring high-flow nasal canula. A CT of the chest showed complete lobar consolidation of the right-lower lobe (RLL) with no change in valve position (Figure 6).

Sputum culture grew methicillin-sensitive *S. aureus*, and the patient was treated with two weeks of cefazolin. After treatment, her symptoms improved, oxygen requirements returned to baseline, and there was no discernable air leak from the chest tube. The tube was removed without issue, and she was discharged home in stable condition with plans for re-implanting a valve in the RML within three-four months.

## Discussion

Our patient suffered lobar pneumonia from methicillin-sensitive *S. aureus* following implantation of four endobronchial valves for severe centrilobular emphysema. Interestingly, the pneumonia developed in the RLL, while the valves were placed in the RUL and RML. Furthermore, it is possible that the pneumonia could have been related to the repeated chest tube placement and bronchoscopy itself. However, given the timeline of events, it is reasonable to assume the pneumonia could have resulted from valve insertion. As previously mentioned, the reported incidence of pneumonia following BLVR varies widely depending on the referenced study. However, little data exists regarding risk factors for development of pneumonia, onset of infection after valve placement, isolated organisms from culture, antibiotics used for treatment, and duration of treatment.

A study performed by Sciurba and colleagues published in 2010 evaluated 214 patients who received endobronchial valves for the management of severe emphysema despite medical management. Within 90 days of the intervention, two patients (0.9%) developed pneumonia distal to the valve, while five patients (2.3%) developed pneumonia that was not distal to the valve [2]. This was not found to be statistically significant compared to the incidence of pneumonia in the control group, which was two patients out of 87 total [2]. There was no mention of isolated organisms from sputum culture, choice of antibiotics, or duration of antibiotics.

A study performed by Herth and colleagues published in 2012 in Europe evaluated 111 patients who received BLVR. Within one year of treatment, seven patients (3.6%) developed pneumonia distal to the valve while 13 patients (11.7%) developed pneumonia that was not distal to the valve [3]. Interestingly, of the patients who developed pneumonia in the non-treated lobe, 50% developed infection in an ipsilateral lobe while the other half developed infection in a contralateral lobe [3]. Overall, the incidence of pneumonia at one year for patients in the treatment group was not statistically significant compared to that in the control group [3]. Again, there was no mention of isolated organisms from sputum culture, choice of antibiotics, or duration of antibiotics.

A study performed by Klooster and colleagues published in 2016 in the Netherlands evaluated 64 patients who received BLVR. Within one year of treatment, five patients (8%) developed pneumonia. Of these patients, 60% developed pneumonia within six months of treatment and 40% developed pneumonia after six months of treatment [4]. Most episodes of pneumonia occurred in the treated lobe (60%) as opposed to the non-treated lobe (40%) [4]. A study performed by Fiorelli and colleagues published in 2017 in Italy reported only one episode of pneumonia out of 33 total patients who received BLVR “that resolved with antibiotic therapy” [5]. Finally, a study performed by Kemp and colleagues published in 2017 in the United States reported six patients who developed pneumonia out of 65 total treated patients (9.2%) within six months of BLVR. Seven patients developed pneumonia (10.8%) within one year of BLVR [6]. Again, there was no mention of isolated organisms from sputum culture, choice of antibiotics, or duration of antibiotics in any of the studies.

Based on this literature review, it is clear that pneumonia is a potential complication following BLVR. However, details regarding causative organisms and proper management remains unclear. Interestingly, some studies indicate that pneumonia is more likely to occur in a non-treated lobe. The mechanism of action regarding infection of a non-treated lobe is unclear but may be related to the bronchoscopic procedure itself as opposed to the presence of a foreign body within the airway. The onset of infection is also variable based on literature review, with some studies reporting increased incidence less than six months from treatment and other studies reporting increased incidence more than six months after treatment. While no studies document the causative organism, clinicians should treat with adequate coverage for common causes of community-acquired pneumonia such as the *Streptococcus* species. Furthermore, if the clinical situation warrants, physicians should consider using antibiotics with adequate coverage for the *Staphylococcus* species. Likewise, duration of treatment should be based on standard management guidelines for community-acquired pneumonia: five-to-seven days of antibiotics is likely adequate, although physicians may decide to treat longer depending on the clinical scenario. Further data is needed regarding additional assessment of potential risk factors for the development of pneumonia in these patients, such as aspiration, use of ICS, presence of immunosuppression, degree of lung function impairment (FEV1), and number and location of valves.

## Conclusions

BLVR has proven efficacy in reducing breathlessness, improving lung function, and improving exercise tolerance in patients with severe heterogeneous emphysema. Physicians should be aware of the potential complications, including pneumothorax, COPD exacerbation, pneumonia, and even death. Pneumonia is seldom reported in patients following BLVR, and rates vary depending on the clinical trial. Treatment strategy varies depending on the clinician but usually mirrors the treatment for community-acquired pneumonia. Despite this, more information is needed regarding specific patient risk factors, causative organisms, choice of antibiotics, and duration of treatment.
